# A Common Model for Cytokine Receptor Activation: Combined Scissor-Like Rotation and Self-Rotation of Receptor Dimer Induced by Class I Cytokine

**DOI:** 10.1371/journal.pcbi.1002427

**Published:** 2012-03-08

**Authors:** Xiaodong Pang, Huan-Xiang Zhou

**Affiliations:** 1 Department of Physics, Florida State University, Tallahassee, Florida, United States of America; 2 Institute of Molecular Biophysics, Florida State University, Tallahassee, Florida, United States of America; Michigan State University, United States of America

## Abstract

The precise mechanism by which the binding of a class I cytokine to the extracellular domain of its corresponding receptor transmits a signal through the cell membrane remains unclear. Receptor activation involves a cytokine-receptor complex with a 1∶2 stoichiometry. Previously we used our transient-complex theory to calculate the rate constant of the initial cytokine-receptor binding to form a 1∶1 complex. Here we computed the binding pathway leading to the 1∶2 activation complex. Three cytokine systems (growth hormone, erythropoietin, and prolactin) were studied, and the focus was on the binding of the extracellular domain of the second receptor molecule after forming the 1∶1 complex. According to the transient-complex theory, translational and rotation diffusion of the binding entities bring them together to form a transient complex, which has near-native relative separation and orientation but not the short-range specific native interactions. Subsequently conformational rearrangement leads to the formation of the native complex. We found that the changes in relative orientations between the two receptor molecules from the transient complex to the 1∶2 native complex are similar for the three cytokine-receptor systems. We thus propose a common model for receptor activation by class I cytokines, involving combined scissor-like rotation and self-rotation of the two receptor molecules. Both types of rotations seem essential: the scissor-like rotation separates the intracellular domains of the two receptor molecules to make room for the associated Janus kinase molecules, while the self-rotation allows them to orient properly for transphosphorylation. This activation model explains a host of experimental observations. The transient-complex based approach presented here may provide a strategy for designing antagonists and prove useful for elucidating activation mechanisms of other receptors.

## Author Summary

Class I cytokines activate their receptors via a 1∶2 complex, but the conformational rearrangements leading to receptor activation remain unclear. To elucidate the activation mechanism, here we calculated the transient complex, an on-pathway intermediate close to the 1∶2 complex. Similar rotational motions were found for three cytokine (growth hormone, erythropoietin, and prolactin) receptors on going from the transient complex to the 1∶2 complex. They involve both scissor-like rotation between the extracellular domains of two receptor molecules and self-rotation of the molecules. Based on these results, we propose a common model for receptor activation by class I cytokines. The model explains a number of experimental observations, including differences in receptor orientations between erythropoietin and its antagonistic and partially agonistic mimetics. Transient complexes present a novel type of targets for designing antagonists. The detailed activation model developed here and our transient-complex based approach will be useful for studying the activation mechanisms of other receptors.

## Introduction

Cytokines are a large family of small proteins that bind to specific cell surface receptors to initiate signals critical for cell proliferation, differentiation, and apoptosis. Among the best characterized cytokines are class I helical cytokines, including growth hormone (GH), erythropoietin (EPO), and prolactin (PRL). Each of these cytokines has two receptor binding sites, referred to as site 1 and site 2, with high and low affinities, respectively. Each cytokine receptor consists of an extracellular domain (ECD) and an intracellular domain (ICD), connected by a single transmembrane helix (TMH). The ECD in turn is composed of two β-sandwich subdomains linked by a short hinge [1]. It is well known that the binding of two receptor molecules, to site 1 and site 2 on the cytokine, results in receptor activation, leading to transphosphorylation of two Janus kinase 2 (JAK2) molecules, each associated with a receptor ICD at a proline-rich region (box 1). Once phosphorylated, the JAK2 molecules initiate downstream signaling [2]–[5].

**Figure 1:**
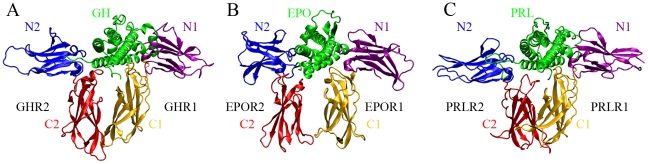
Structures of the 1∶2 complexes. (A) GH:(GHR)_2_. (B) EPO:(EPOR)_2_. (C) PRL:(PRLR)_2_. The cytokine (GH, EPO, or PRL) is in the middle in green; receptor 2 (R2) is on the left and receptor 1 (R1) is on the right. Each receptor ECD is composed of an N-terminal subdomain (N1 in purple and N2 in blue) and a C-terminal subdomain (C1 in orange and C2 in red).

The structures of the 1∶2 complexes of GH, EPO, and PRL with the ECDs of the corresponding receptors have been determined [1], [6], [7] (Figure 1). The structures are overall similar, but differ in many details. Each cytokine contacts both ECD subdomains of each receptor molecule around the hinge. The two C-terminal subdomains are nearly parallel to each other (and presumably to the normal of the cell membrane), while the two N-terminal domains lie on a plane parallel to the membrane, at 130°–160° angles. These structures have been very valuable, but they do not reveal the rearrangement of the two ECDs induced by the cytokine binding. Since the structures lack the TMHs and the ICDs, there is also no information on the ICDs' rearrangement, which initiates downstream signaling. The aim of the present study is to compute the cytokine-induced rearrangement of the ECDs and develop a detailed model for receptor activation.

In the early model proposed by Fuh et al. [8] for GH receptor activation, GH first binds to one receptor molecule via site 1, and then recruits the second receptor molecules via site 2. This sequential receptor-dimerization model was based on three important observations. First, site 1 has much higher affinity than site 2. Second, a G120R mutation disrupting site 2 did not affect receptor binding to site 1 but abolished GH-induced cell proliferation. Third, the dose response curve of cell proliferation was bell-shaped, suggesting that engagement of each receptor molecule by a separate GH molecule (via site 1) interferes with receptor dimerization and signaling.

It is now clear that receptors likely exist as preformed dimers in the absence of the cytokines [9]–[11]. For both GH receptor (GHR) and EPO receptor (EPOR), the TMHs are implicated in dimer formation [10], [12], [13]. However, dimerization alone is insufficient for activation. For example, two EPO mimetic peptides (EMP1 and EMP33) bind to EPOR to form 1∶2 complexes, but in each of these complexes the ECDs (and their subdomains) have an orientational arrangement that is different from that in the EPO:(EPOR)_2_ complex [6], [14], [15]. (EMP1 and EMP33 each are present as dimers in the complexes with two EPORs. We treat these dimers as a single ligand and refer to the stoichiometry of the complexes as 1∶2.) In signaling EMP1 acted as a partial agonist but EMP33 as an antagonist. Seubert et al. [16] engineered EPOR dimers by replacing the ECDs with a dimeric coiled coil. Through deletions of up to 6 residues, they explored the full range of relative orientation of the two TMHs in the EPOR dimers, and found one of them to be constitutively active in cell proliferation.

For GHR, Rowlinson et al. [17] found monoclonal antibodies that competed against GH for GHR binding but failed to act as agonists, again indicating that dimerization is insufficient for activation. Brown et al. [10] demonstrated constitutive dimer formation of GHR by FRET experiments, and after inserting alanine residues in the TMH or in the sequence immediately before box 1, observed constitutive activity. Interestingly, constitutive activity required different numbers of inserted alanine residues in the TMH and before box 1.

The deletion and insertion results of Seubert et al. [16] and Brown et al. [10] suggest that rotation of the TMH is involved in receptor activation. However, the orientational rearrangement of the ECDs that is induced by cytokine binding and triggers the TMH rotation remains unclear.

Even in binding to a preformed dimer, it is still believed that engagement of site 1 precedes engagement of site 2 [5], [10], [18]. The initial step, i.e., the binding of a cytokine to the first receptor molecule (R1) via site 1, leads to a 1∶1 complex. The 1∶1 complex is very likely an on-pathway intermediate since the structures of the 1∶1 complexes formed by GH and GHR ECD [19], [20] and by PRL and PRL receptor (PRLR) ECD [21] are very similar to those in the corresponding 1∶2 complexes [1], [7], [22]. The 1∶1 complexes were obtained by introducing the site-2 disrupting mutation G120R to GH and a corresponding mutation, G129R, to PRL.

Recently we calculated the rate constants for forming the 1∶1 complexes of PRL, GH, and EPO [23], using our transient-complex theory [24]. These rate constants differ by 5000-fold, mostly arising from differing levels of charge complementarity across the site-1 interface. Moreover, the rate constants of the initial binding apparently anti-correlate with the circulation concentrations of the cytokines, such that the pseudo-first order receptor binding rate constants are close to the limits set by the half-lives of the receptors, ensuring their participation in cytokine binding before internalization and degradation.

The transient complex in a binding process refers to an intermediate that has near-native relative separation and orientation but not the short-range specific interactions of the native complex, and is formed by translational and rotational diffusion of the subunits. The transient complex is located at the rim of the energy well of the native complex, and is therefore a late on-pathway intermediate. Structural differences between the transient complex and the native complex reveal the orientational rearrangement of the subunits at the late stage of the binding process. This stage starts after some of the native contacts are already in proximity, but before the precise fit of all the native contacts. As such it is at a critical juncture of the binding process. Yet its characterization enjoys certain technical advantages. First, because we focus on the late stage, we completely avoid any issues concerning how the subunits reach the transient complex, such as 2-dimensional diffusion of the membrane-bound receptors. Second, because the transient complex is formed before the formation of the stereospecific native contacts, we also avoid the necessity of accurately treating the native contacts. Instead, the transient-complex ensemble is largely dictated by the shape of the binding interface.

Here we applied the transient-complex theory to study the binding of a second receptor molecule (R2) to a 1∶1 complex, to form the 1∶2 activation complex. By calculating the transient complex for this step, we identified the orientational rearrangement between the ECDs of R1 and R2 leading to receptor activation. Similar rotational motions were found for three cytokine-receptor systems (GH, EPO, and PRL with their receptors). At the start of the late-stage orientational rearrangement, R2 is loosely bound to the 1∶1 complex around site 2 of the cytokine, with the C-terminal subdomains of R1 and R2 far apart. R1 and R2 then rotate like a scissor, around an axis along the N-terminal subdomain of R2, to close up the membrane-proximal ends of the two C-terminal subdomains. In addition, R1 and R2 both self-rotate but to different extents, such that the angle between the two N-terminal subdomains is reduced. We propose that the scissor-like rotation separates the intracellular domains of the two receptor molecules to make room for the associated Janus kinase molecules, while the self-rotation allows them to orient properly for transphosphorylation. This common model for receptor activation explains a host of experimental observations on the three cytokine-receptor systems.

## Results/Discussion

The focus of the present study is the late-stage orientational rearrangement between the two receptor molecules in forming the 1∶2 complex. The start of the late stage is the transient complex, in which R2 is loosely bound to the 1∶1 complex around site 2 of the cytokine. The transient complex is identified by mapping the energy landscape over the native-complex energy well and the surrounding region, using the structure of the native complex as input [24], [25]. Within the native-complex well, the rotational freedom of the subunits is severely restricted. As the two subunits separate, there is a sudden increase in the rotational freedom. The transient complex is identified with the midpoint of this transition, which is largely dictated by the shape of the binding interface.

Receptor activation occurs at cell membranes, where receptors likely exist as preformed dimers. However, the rate constants for binding to the 1∶1 complex by R2 ECD coming from the bulk solution, rather than from a preformed receptor dimer, have been measured for the GH-GHR and PRL-PRLR systems [22], [26]. Our transient-complex theory can make accurate predictions for the rate constants of protein association in bulk solution, as demonstrated by results spanning five orders of magnitude for 49 protein complexes [25]. We carried out rate constant calculations for the 1∶2 complexes of the three cytokines with the corresponding receptor ECDs. The results were within the range, 10^4^ to 10^6^ M^−1^ s^−1^, of the *in vitro* measurements (see Supporting Text S1 for details and implication for R2 binding to the 1∶1 complex in the cellular environments).

### Transient complexes of three cytokine-receptor systems

Each transient complex was an ensemble of configurations located at the rim of the native-complex energy well. It was generated from the structure of the 1∶2 complex and would be a late on-pathway intermediate, even if R2 came from a preformed receptor dimer.

As noted above, the transient complex was identified by mapping the energy landscape over the native-complex energy well and the surrounding region. The internal conformations of R2 and the 1∶1 complex (referred to as two subunits) were fixed at those in the 1∶2 native complex. This is justified since the available structures of the isolated 1∶1 complexes of the GH and PRL systems [19]–[21] are very similar to those in the corresponding 1∶2 complexes [1], [7], [22] (with C_α_ RMSDs of ∼1.2 Å); similarly the structures of apo GHR [10] and of apo EPOR [9] as well as EPORs in EMP1:(EPOR)_2_ and EMP33:(EPOR)_2_ [14], [15] are similar to the R2 structures in the respective 1∶2 complexes for GH and EPO (with C_α_ RMSDs of ∼1.3 Å). In particular, there is no evidence for significant change in the relative orientation between the N-terminal and C-terminal subdomains of either ECD upon forming any 1∶2 complex. (Calculations using some of these alternative structures as well as those taken from molecular dynamics simulations of the 1∶2 complexes produced similar results.) There were then only six remaining degrees of freedom in mapping the inter-subunit energy landscape: three for relative separation and three for relative rotation.

**Figure 2:**
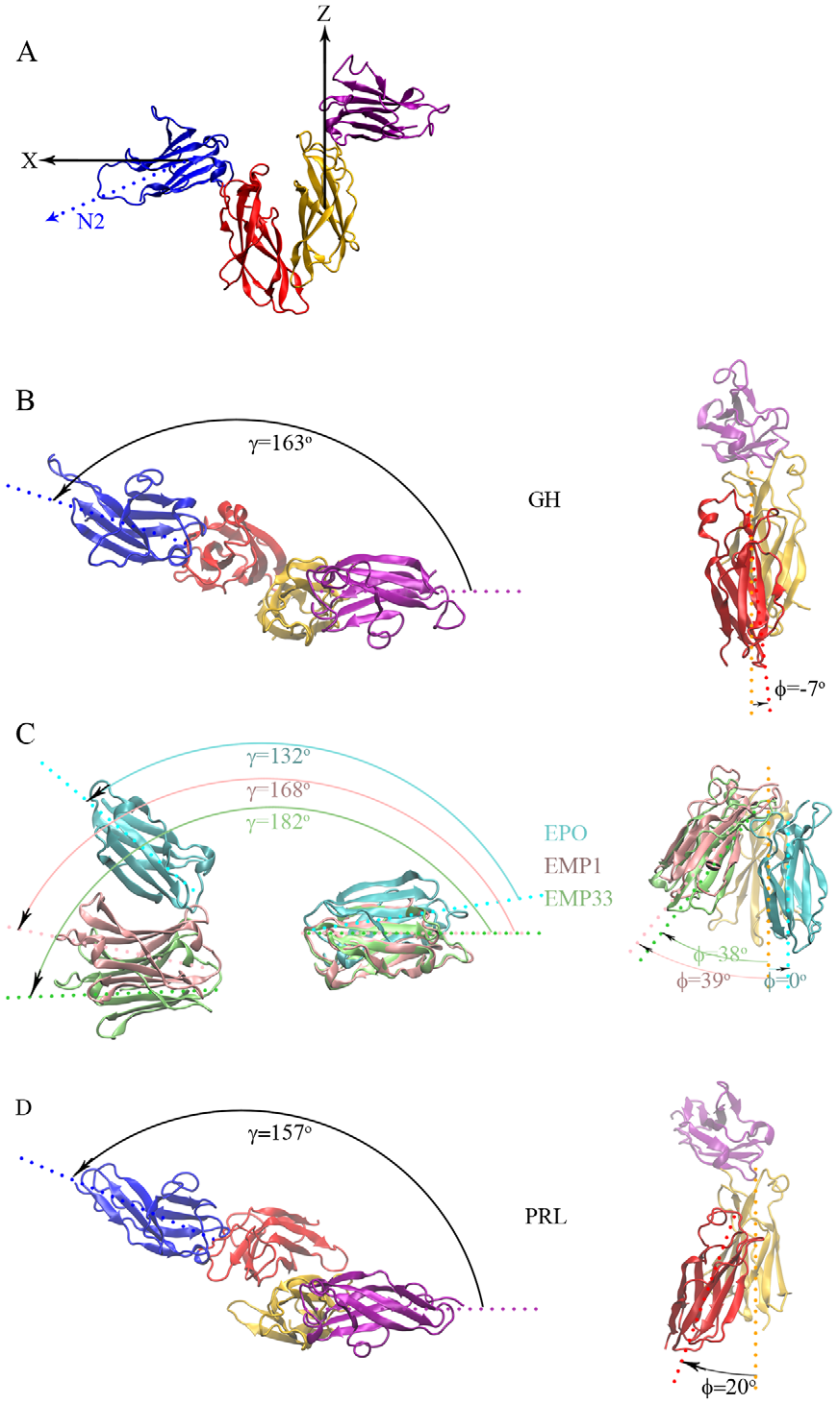
Top and side views of the relative orientations between R1 and R2 in the 1∶2 complexes. Cytokines are removed for clarity. (A) A coordinate system for defining the top and side views. The long axis of the C1 subdomain, identified with the principal axis corresponding to the largest moment of inertia, was chosen as the *z* axis (pointing upward). The *x* axis was chosen to be in the plane defined by the long axes of the C1 and N2 subdomains. This coordinate system is illustrated by the (GHR)_2_ complex, viewing into the *y* axis. Top (left) and side (right) views of (GHR)_2_, (EPOR)_2_, and (PRLR)_2_ are displayed in (B)–(D), with the cytokine names listed in the middle. In (B) and (D) side views, N2 is not displayed. In (C), C1 and C2 are not displayed in the top view, and N1 and N2 are not displayed in the side view. The coloring scheme in (A), (B), and (D) is the same as in Figure 1. In (C) the two EPORs in EMP1:(EPOR)_2_ and EMP3:(EPOR)_2_ are also displayed; receptors in complex with EPO, EMP1, and EMP33 are displayed in cyan, pink, and lime green, respectively (except that C1 in the side view is in orange). The values of *γ* and *ϕ* angles are also shown.

To facilitate describing the orientational rearrangement on going from the transient complex to the native complex, we refer to the N-terminal and C-terminal subdomains of the R1 ECD as N1 and C1, and analogously N2 and C2 for the subdomains of R2. We present orientational changes as rotations of R2 relative to R1. To that end, we define a coordinate system in which the *z* axis is the long axis of C1 (directed upward), the *y* axis is perpendicular to the long axes of C1 and N2, and consequently the *x* axis is in the plane defined by the two long axes and roughly parallel to the N2 long axis (Figure 2A). We refer to the view into the *z* axis as top view, and the view into the *x* axis as side view. Figure 2B–D presents the configurations of the receptor molecules in the 1∶2 native complexes of the three systems in these two viewing directions.

**Figure 3:**
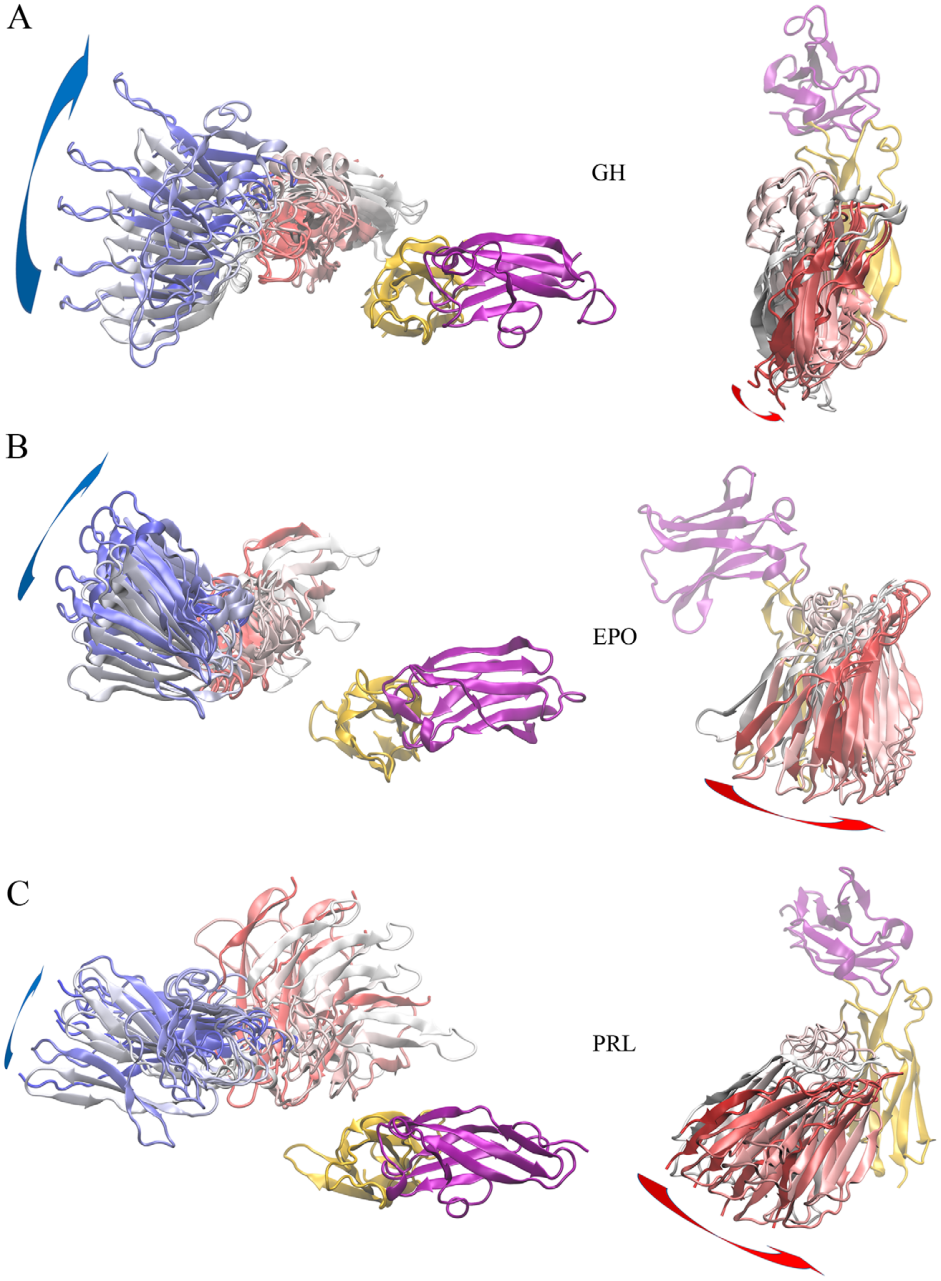
Representative configurations of the transient complexes. (A)–(C) Top views are on the left, side views are on the right, and cytokine names are listed in the middle. R1 is shown with N1 in purple and C1 in orange. Each R2 configuration is displayed with color varying from blue at the N-terminal to red at the C-terminal. N2 is not shown in the side views.

In Figure 3 we display 5 representative transient-complex configurations each for the GH-GHR, EPO-EPOR, and PRL-PRLR systems. The top view shows that, for each of the three systems, R2 undergoes clockwise rotation around the *z* axis on going from the transient complex to the 1∶2 native complex. This “self-rotation” is most prominent for N2 and less so for C2, since the latter is roughly parallel to the rotation axis (i.e., *z* axis). Meanwhile the side view shows that, again for each of the three systems, R2 undergoes counterclockwise rotation around the *x* axis on going from the transient complex to the 1∶2 native complex. This “scissor-like rotation” brings together the membrane-proximal ends of C1 and C2.

To quantitatively characterize the orientational rearrangement, we define two angles: *γ* for the angle between the projections of the N1 and N2 long axes on the *x*-*y* plane; and *ϕ* for the angle between the projections of the C1 and C2 long axes on the *y*-*z* plane. The values of these angles in the native complexes of are: *γ* = 163° and *ϕ* = −7° in GH:(GHR)_2_; *γ* = 132° and *ϕ* = 0° in EPO:(EPOR)_2_; and *γ* = 157° and *ϕ* = 20° in PRL:(PRLR)_2_ (Figure 2B–D). From the transient complex to the native complex, clockwise self-rotation can be recognized as a decrease in *γ*, and scissor-like rotation can be recognized as a decrease in *ϕ*. The distributions of *γ* and *ϕ* in the transient complexes of the three systems are shown in Figures S1, S2, and S3. The distributions are asymmetric with respect to the *γ* and *ϕ* values in the native complexes, with higher values more favored in the transient complexes, supporting the self-rotation and scissor-like rotation illustrated in Figure 3 on going from the transient complex to the native complex.

Mark and co-workers [27], [28] carried out molecular dynamics simulations of (GHR)_2_ after removing GH from its 2∶1 complex and of (PRLR)_2_ after removing PRL from its 2∶1 complex. In the former simulations they found prominent self-rotation corresponding to that depicted in the top view of Figure 3A. In the latter simulations they found prominent scissor-like rotation corresponding to that depicted in the side view of Figure 3C. The simulation results thus accord well with our transient-complex calculations.

Examination of the structures of the three 1∶2 native complexes revealed that the asymmetry in *ϕ* can be attributed to the wrapping of a C1 loop (between strands A and B) around C2 (Figure S4). A C2 configuration with *ϕ* lower than the native value tends to encounter steric clash with the C1 loop. In contrast, C1 presents a relatively flat surface on the side of the native C2 where *ϕ* is higher than the native value, allowing the sampling of the high *ϕ* values. In the cases of GH:(GHR)_2_ and PRL:(PRLR)_2_, the extended N-terminal tail of the cytokine enforces the asymmetry in *γ* by providing an additional interaction surface for N2 configurations with *γ* higher than the native value. Recent experimental results of Jomain et al. [21] have implicated a role of the PRL N-terminal tail in receptor activation. The dictation of the transient-complex ensemble by the interface shape is reminiscent of observations on the binding of a ribotoxin to an RNA loop on the ribosome [29]; there ribosomal proteins around the binding interface were found to shift the positioning of the transient-complex ensemble.

### Receptor activation model combining scissor-like rotation and self-rotation

Our transient-complex calculations revealed the ECD orientational rearrangements of the three receptor dimers induced by the binding of the corresponding cytokines. These orientational rearrangements are similar, involving both self-rotation and scissor-like rotation, and are largely dictated by the shape of binding interface.

**Figure 4:**
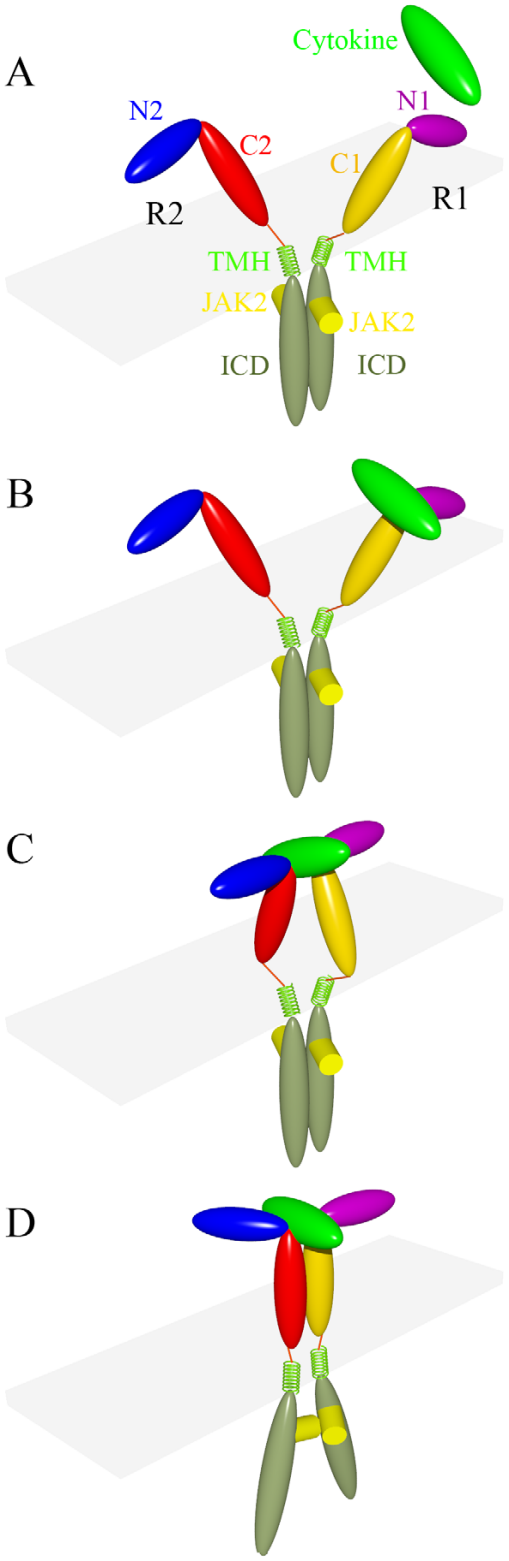
Model for receptor activation. The cytokine and ECDs of the two receptor molecules are colored in the same scheme as in Figure 1. The ECD-TMH linkers are represented by red lines, and the TMHs are represented by lime green coils. Residue Cys241 in GHR is at the midpoint of the ECD-TMH linker. The ICDs are in turquoise green and JAK2s are in yellow. (A) In the preformed dimer, the two ECDs are apart, but the TMHs and ICDs are in contact; the associated JAK2s are oriented away from each other. (B) The cytokine binds to R1 via site 1 to form the 1∶1 complex. (C) R2 approaches site 2 of the cytokine in the 1∶1 complex, resulting in a loose complex in which the N-terminal subdomains of the two ECDs are nearly anti-parallel and the membrane-proximal ends of the two C-terminal subdomains are apart. (D) Scissor-like rotation of R1 and R2 leads to separation of the ICDs, making room for the JAK2s to approach each other. Self-rotation of R2 (and R1 to a lesser extent) further allows the JAK2s to orient properly for transphosphorylation.

The orientational rearrangement of the ECDs has to be transmitted via the TMHs to the ICDs, to properly position and orient the associated JAK2 molecules for transphosphorylation. Based on our previous study [23] and the present results on the three cytokine-receptor systems, we propose a common model for receptor activation illustrated in Figure 4 (see also Supplementary Video S1). First a cytokine binds to an unoccupied receptor R1 via site 1 to forms a 1∶1 complex. Then R2 in the preformed dimer approaches site 2. Initially the ECD N-terminal subdomains of R1 and R2 are separated at ∼180° and the membrane-proximal ends of the two ECD C-terminal subdomains are apart. Subsequently the two ECDs undergo scissor-like rotation to bring together the membrane-proximal ends of the two C-terminal subdomains, and simultaneously self-rotation to reduce the angle between the N-terminal subdomains. As a result of the scissor-like rotation, the ECD-TMH linkers and the N-terminals of the TMHs move closer, while the C-terminals of the TMHs and the box-1 regions of the ICDs are separated, making room for the associated JAK2 molecules. Meanwhile the self-rotation allows the JAK2 molecules to orient properly for transphosphorylation.

Our calculations were based on the structures of the 1∶2 complexes of the three cytokines with the corresponding receptor ECDs. These structures are likely preserved in the 1∶2 complexes involving the full-length receptors bound to cell membranes, for the following reasons. First, structures of the receptor ECDs in apo form and in 1∶1 and 1∶2 complexes with their cytokines have been determined by different groups. As noted above, the multiple structures for each system are all very similar, attesting to their stability. Second, the ECD of each receptor is separated from the TMH by a linker of ∼10 residues, suggesting minimal perturbation of the ECD by the TMH in the full-length receptor. While separating the ECDs from the TMHs, the linkers play the important role of relaying the rotational motions of the ECDs to the TMHs. (A similar role was identified for an inter-domain linker in the activated of a ligand-gated ion channel [30].)

The ECD orientational rearrangements of the receptor dimers determined here occur after the two receptor molecules are loosely bound, and thus the fact that the molecules reach this state via diffusion in the 2-dimensional membrane has no bearing. The resulting motions of the TMHs and box-1 regions are speculated, but seem to be supported by a host of experimental observations, as we detail below.

### Incomplete rotation leads to partial agonist or antagonist

Our transient-complex calculations identified a common rotational pathway that receptor dimers are likely to follow upon ligand binding. If the rotations induced are incomplete, then the ligand will likely act as a partial agonist or antagonist. This conclusion is supported by the EPOR partial agonist EMP1 and antagonist EMP33. In EMP1:(EPOR)_2_, *γ* = 168° and *ϕ* = 39° (Figure 2C). Both values are higher than the counterparts in EPO:(EPOR)_2_, just like those in the transient complex of EPO:(EPOR)_2_ (Figure S2). That is, in terms of receptor orientational arrangement, EMP1:(EPOR)_2_ and the transient complex of EPO:(EPOR)_2_ deviate from EPO:(EPOR)_2_ from the same direction. The receptor configuration induced by EMP1 can thus be viewed as an intermediate along the way to the fully activated configuration as found in EPO:(EPOR)_2_, explaining why EMP1 is only a partial agonist. In EMP33:(EPOR)_2_, *γ* = 182° and *ϕ* = 38° (Figure 2C), the former angle deviating even more than that in EMP1:(EPOR)_2_ from the counterpart in EPO:(EPOR)_2_. The receptor configuration induced by EMP33 is thus an earlier intermediate compared to that induced by EMP1, and hence EMP33 is an antagonist. The fact that EMP1 is a partial agonist but EMP33 is an antagonist despite the similar *ϕ* angles of EMP1:(EPOR)_2_ and EMP33:(EPOR)_2_ directly supports our contention that both scissor-like rotation and self-rotation are required for receptor activation (see below for further discussion).

We also calculated the transient complexes formed by EMP1 and EMP33 with EPOR, and found that they too followed the common rotational pathway of the GH-GHR, EPO-EPOR, and PRL-PRLR systems. The distributions of *γ* and *ϕ* for the EMP1 and EMP33 transient-complex ensembles are shown in Figure S2. Figure S5 displays 5 representative configurations each for the EMP1 and EMP33 transient complexes. Clockwise self-rotation (top view) and scissor-like rotation (side view) similar to those shown in Figure 3 are also seen in approaching the native complexes here.

From the distributions of *γ* and *ϕ* in Figure S2, it can seen that the EMP33 transient complex is comprised of configurations closely clustered around the EMP33 native complex, and they all fall inside the configurational space of the EMP1 transient complex. It appears that EMP33 locks the receptor dimer in the configurations found in the EMP1 transient complex and prevents it from further orientational rearrangement toward more active configurations. EMP33 differs from EMP1 by two additional bromine atoms on Tyr4 residues (located in site 1 and site 2) of the dimeric ligand. The additional contacts seem key to the locking action of EMP33.

Our analysis on the complexes of EMP1 and EMP33 with EPOR suggests a strategy for designing antagonists based on transient-complex calculations. One first uses the configurations constituting the transient complex of a full agonist as targets; ligands (like EMP1) that stabilize these transient-complex configurations may be candidates for partial agonists. In the next iteration, configurations constituting the transient complex of a thus designed partial agonist become targets; ligands (like EMP33) that stabilize the new generation of transient-complex configurations may be candidates for antagonists. This process may be further iterated.

### Both scissor-like rotation and self-rotation are required for activation

Constitutively active receptors obtained by Seubert et al. [16] and Brown et al. [10] through deletion or insertion mutations on TMHs demonstrate the involvement of self-rotation in receptor activation. Insertions and deletions move residues on the C-terminal side of the point of mutation along the helical wheel. This has the same effect as self-rotation on the associations JAK2s. Each deleted (inserted) residue in the TMH corresponds to a 103° counterclockwise (clockwise) rotation (top view). Starting with the state in which R2 is loosely bound to the 1∶1 complex (Figure 4C), we find that, after either deleting three residues or inserting four residues on the TMHs, the associated JAK2s are oriented in proximity (Figure S6), similar to that brought about by the receptor self-rotation in our activation model (Figure 4D). These are precisely the numbers of deleted and inserted residues that Seubert et al. [16] and Brown et al. [10] found to result in constitutive activity. We emphasize, however, both self-rotation and scissor-like rotation are required in our model of receptor activation. We note that the dimeric coiled coil replacing the ECDs in the constitutively active EPOR mutant engineered by Seubert et al. [16] would likely bring the N-terminals of the TMHs together, thus achieving the same effect as cytokine-induced scissor-like rotation.

Other experimental observations also support the proposed role of scissor-like rotation in receptor activation. Zhang et al. [31] found that a disulfide linkage between Cys241 residues, located in the middle of the ECD-TMH linkers (Figure 4), occurred only after forming the GH:(GHR)_2_ complex. This observation suggests that the ECD-TMH linkers are apart before GH binding and come into contact in the 1∶2 complex. This movement of the linkers is just what is brought about by the scissor-like rotation of R1 and R2 (Figure 4).

Brooks et al. [32] using FRET observed that GHR ICDs moved part by ∼9 Å in an active receptor dimer relative to an inactive dimer. They concluded that reorientation (akin to our self-rotation) is critical but insufficient for full activation. Their observation and conclusion are in line with our model of receptor activation.

Recently Liu and Brooks [33] replicated the alanine-insertion of Brown et al. [10] on PRLR. In contrast to the results of Brown et al. for GHR, Liu and Brooks did not find any constitutively active dimer after inserting up to four alanines. Since it takes seven residues to cover all positions on a helix wheel, insertions of five and six alanines would be required to complete the full range of relative orientation of the two TMHs. It is possible that the five- or six-alanine insertion mutant would be constitutively active. It is also possible that none of these alanine-insertion PRLR mutants has sufficient scissor-like rotation for activation.

Other experiments can be designed to further test our model of cytokine receptor activation. For example, inter-receptor distances at different positions along the *z* axis could be obtained by double cross-linking with bifunctional reagents, which bridge between two receptor molecules and can be used as molecular rulers [34]. The distances, before and after cytokine binding, between residues in the ECD-TMH linkers and between residues in the box-1 regions will be particularly useful for validating and refining our model. It will then even be worthwhile to start building structural models for receptor constructs that are truncated only after the box-1 region, as either preformed dimer or in an activated complex.

Orientational rearrangements such as self-rotation have been implicated in the activation of thrombopoietin receptor and many tyrosine kinase receptors [35]–[37]. The detailed activation model presented here for three cytokine receptors and our approach based on transient-complex calculations will be useful for elucidating the activation mechanisms of a wide range of receptors.

In conclusion, our calculations suggest that R2 undergoes a combined scissor-like rotation and self-rotation to reach the activated state upon binding to the cytokine-R1 complex. The similar observations in all the three cytokine-receptor systems allow us to propose a common model for class I cytokine receptor activation. Both the scissor-like and self-rotation are required for the activation

## Methods

### Structure preparation for native complexes

The implementation of our transient-complex theory used the structures of native complexes as input. Here native complex referred to a 1∶2 complex comprised of one cytokine molecule and two receptor molecules. The structures of the 1∶2 complexes of the GH-GHR, EPO-EPOR, PRL-PRLR, EMP1-EPOR, and EMP33-EPOR systems were from Protein Data Bank entries 3HHR [1], 1EER [6], 3NPZ [7], 1EBP [14], and 1EBA [15], respectively. In the complex containing either EMP1 or EMP33, the EPO mimetic peptide was present as a dimer. All hydrogen atoms were added and energy minimized by the AMBER program.

The N-terminal tail of GH (residues 1 to 5) changes orientation on going from the 1∶1 complex to the 1∶2 complex, from extending sideways to wrapping around R2. We used the orientation of the N-terminal tail of GH in the 1∶1 complex, but counted those N-terminal residues in touch with R2 in the 1∶2 complex when calculating contacts for determining the transient complex (see below).

The N-terminal tail of PRL (residues 1 to 10) is disordered in both the 1∶1 and 1∶2 complexes, and shows an ensemble of conformations in the NMR structure of the unbound state (Protein Data Bank entry 1RW5) [38]. Jomain et al. [21] implicated a role of the N-terminal tail in receptor activation. We thus chose to build the N-terminal tail by Modeller (version 9v8) [39], in an orientation wrapping R2 and similar to that in one of the NMR models for the unbound PRL. To further mimic the situation with the GH-GHR system, we pulled the N-terminal tail so that it extended sideways. The subsequent treatment of this N-terminal tail when determining the transient complex was the same as described for the GH-GHR system.

### Implementation of the transient-complex theory

The implementation of our transient-complex theory for protein-protein association has been described previously [23]–[25]. Briefly, while fixing the 1∶1 complex in space, R2 was translated and rotated around the native-complex configuration. The three translational degrees of freedom were represented by the displacement vector **r** between the centers of the binding surfaces on the two subunits. The binding surfaces were defined by heavy atoms making <5 Å cross-interface contacts in the native complex. Of the three rotational degrees of freedom, two were a unit vector **e** attached to the mobile R2 and the remaining one was the rotational angle *χ* around the unit vector. The unit vector was perpendicular to the least-squares plane of the interface heavy atoms.

To sample the native-complex energy well and the transition region to the unbound state, the six translational and rotational coordinates (**r**, **e**, *χ*) were randomly generated, with the magnitude, *r*, of **r** restricted: *r*≤*r*_cut_. The value of *r*_cut_ was automatically determined to ensure that the clash-free fraction of the randomly generated configurations was ≥10^−4^ [25]. The resulting *r*_cut_ values were 6, 6, 12, 6, and 7 Å for the GH-GHR, EPO-EPOR, PRL-PRLR, EMP1-EPOR, and EMP33-EPOR systems, respectively. Clash between the 1∶1 complex and R2 was detected exhaustively over all inter-subunit atom pairs.

For each clash-free configuration, the total number, *N*_c_, of contacts, either native or nonnative, made by a list of “interaction-locus” atoms across the binding interface was calculated as a surrogate of short-range interaction energy. The interaction-locus atoms were selected from the interface atoms as follows. Native pairs of the interface heavy atoms were sorted in ascending order of interatomic distances; each pair was then evaluated against preceding pairs for possible elimination. Specifically, a pair was eliminated if it was within 3.5 Å of a preceding pair on either side of the interface. The final remaining list constituted the interaction-locus atoms. The purpose of the selection process was twofold: to increase the chance that retained native pairs were distinct from each other; and to decrease the chance of nonnative contacts so that there was a proper balance between native and nonnative contacts. The value of *N*_c_ in a randomly generated configuration was calculated by counting the number of native contacts and nonnative contact. The upper limit in distance for forming a native contact was the native distance plus 3.5 Å. To count nonnative contacts, the native distance of each native pair was split in half to define the contact radii of the two atoms. A nonnative contact was considered formed when the interatomic distance was less than the sum of their contact radii plus 2.5 Å.

The *N*_c_ values of the GH-GHR, EPO-EPOR, PRL-PRLR, EMP1-EPOR, and EMP33-EPOR native complexes were 56, 32, 81, 31 and 44, respectively. As the two subunits moved apart, *N*_c_ decreased gradually and the range of allowed rotation angles, as indicated by the standard deviation in *χ* of the clash-free configurations, increased sharply. The midpoint of this sharp transition (where *N*_c_≡*N*_c_*) defined the transient complex (Figure S7) [25]. From 8×10^6^ clash-free configurations, the values of *N*_c_* were determined to be 12, 15, 16, 19, and 13, respectively, for the five systems, and the 9,114, 48,078, 2,276, 19,407, and 13,361 configurations with these respective *N*_c_ values constituted the transient-complex ensembles.

By calculating the basal rate constant to reach the transient complex and the electrostatic interaction energy within the transient complex, the transient-complex theory further predicts the protein association rate constant in solution. Details of these two components and the calculated rate constants are given in Supporting Text S1.

## Supporting Information

Figure S1 Histograms of *γ* and *ϕ* angles of the GH:(GHR)_2_ transient complex. 6,354 transient-complex configurations are used for calculating the histograms. Vertical dashed lines indicate *γ* and *ϕ* values in the native complex.
(TIF)

Figure S2 Histogram of *γ* and *ϕ* angles of the EPO:(EPOR)_2_, EMP1:(EPOR)_2_, and EMP33:(EPOR)_2_ transient complexes. 4,442, 5,760, and 5,994 configurations, respectively, are used for calculating the histograms of the three systems. Vertical dashed lines indicate *γ* and *ϕ* values in the native complexes.
(TIF)

Figure S3 Histogram of *γ* and *ϕ* angles of the PRL:(PRLR)_2_ transient complex. 2,276 transient-complex configurations are used for calculating the histograms. Vertical dashed lines indicate *γ* and *ϕ* values in the native complex. The two peaks in the *γ* histogram, to the right and left of the native value, correspond to R2 configurations forming contact mainly with the N-terminal tail and with the rest of the cytokine, respectively.
(TIF)

Figure S4 The role of a loop in the C1 subdomain in determining the asymmetric distribution of the transient-complex ensemble. This loop, illustrated here in blue on the GH-GHR system, is between strands A and B. GH and R1 are in gray; native R2 is in lime green; and a R2 configuration in the transient complex is in red. An R2 configuration with a *ϕ* angle lower than the native value would have its C2 subdomain positioned toward the foreground of the present view and would likely clash with the C1 loop. In contrast, the R2 configuration shown in red has a *ϕ* angle higher than the native value, and the C2 subdomain, positioned toward the background, is opposite to a flat surface of C1. The extended N-terminal (in yellow) of GH “attracts” R2 configurations (such as the one shown in red) with *γ* angles higher than the native value.
(TIF)

Figure S5 Representative configurations of the transient complexes of two EPO mimetic peptides. (A) EMP1:(EPOR)_2_. (B) EMP33:(EPOR)_2_.
(TIF)

Figure S6 Reorientation of JAK2s resulting from deleting or inserting TMH residues. Top view of the ICDs and the associated JAK2s are shown; a shaded rectangle represents the cell membrane. An α-helix has ∼3.5 residues per turn, so each residue spans ∼360°/3.5 = 103° of the helical wheel. (Left) Deleting each TMH residue would rotate the associated JAK2 counterclockwise for 103°; (Right) Inserting each TMH residue would rotate the associated JAK2 the same amount but in the opposite direction. Negative and positive numbers indicate the total numbers of deleted and inserted TMH residues. Shown in highlight are the three-residue deletion and four-residue insertion, both of which orient the two JAK2 in proximity, ready for transphosphorylation.
(TIF)

Figure S7 Locating the transient complex. (A) GH:(GHR)_2_. (B) EPO:(EPOR)_2_ (C) PRL:(PRKR)_2_. σ_χ_ represents the standard deviation of the χ angles sampled by the clash-free configurations at a given *N*_c_. Symbols represent raw data from the randomly generated clash-free configurations; curve represents the fit to a function used for modeling protein denaturation data as two-state transition. The “baseline” with low σ_χ_ (and high *N*_c_) correspond to configurations in the native-complex well. The “baseline” with high σ_χ_ (and low *N*_c_) correspond to the start of the unbound state. The midpoint of the transition, where *N*_c_ is designated *N*_c_*, identifies the transient complex.
(TIF)

Text S1 Methods for calculating association rate constants in solution and results.
(DOC)

Video S1 Video illustrating our model for receptor activation.
(AVI)
