# Supplementary figures and images for: A Common Model for Cytokine Receptor Activation: Combined Scissor-Like Rotation and Self-Rotation of Receptor Dimer Induced by Class I Cytokine
Source: PLoS Comput Biol. 2012 Mar 8;8(3):e1002427. doi: 10.1371/journal.pcbi.1002427 (PMC3297564; doi:10.1371/journal.pcbi.1002427)

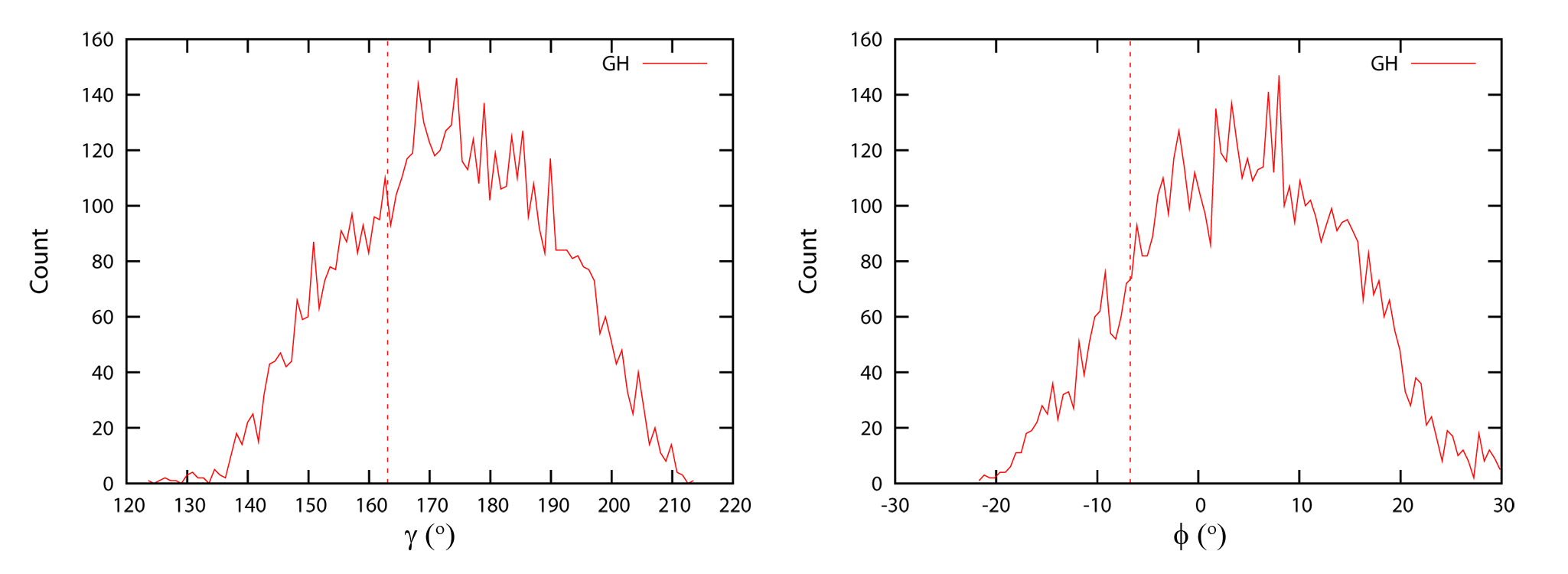

Supplement: Figure S1 — Histograms of γ and ϕ angles of the GH:(GHR)2 transient complex. 6,354 transient-complex configurations are used for calculating the histograms. Vertical dashed lines indicate γ and ϕ values in the native complex. (TIF) [file pcbi.1002427.s001.tif]

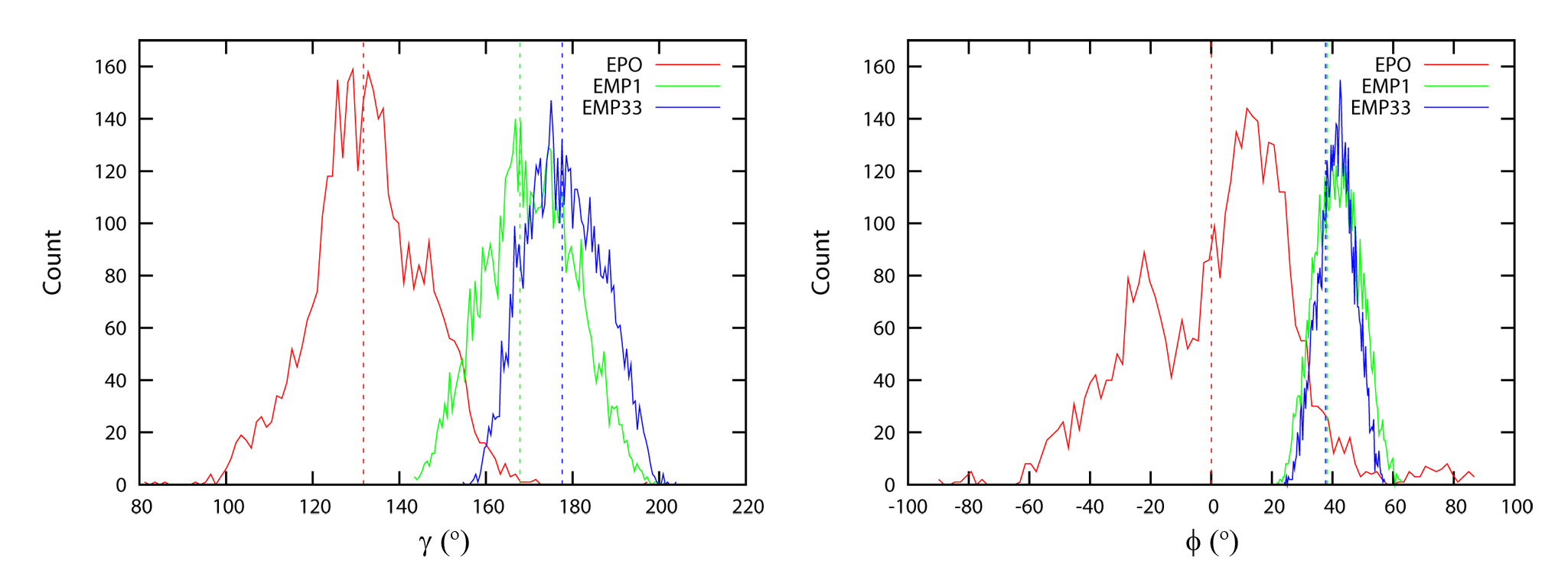

Supplement: Figure S2 — Histogram of γ and ϕ angles of the EPO:(EPOR)2, EMP1:(EPOR)2, and EMP33:(EPOR)2 transient complexes. 4,442, 5,760, and 5,994 configurations, respectively, are used for calculating the histograms of the three systems. Vertical dashed lines indicate γ and ϕ values in the native complexes. (TIF) [file pcbi.1002427.s002.tif]

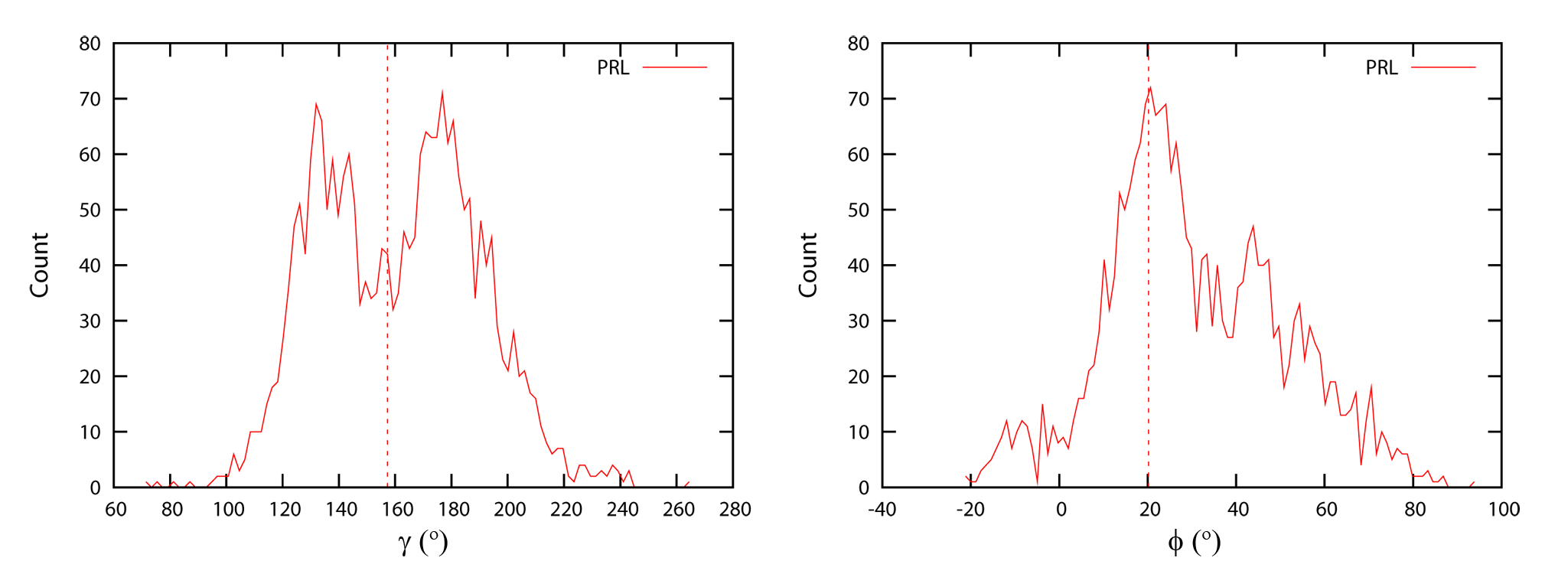

Supplement: Figure S3 — Histogram of γ and ϕ angles of the PRL:(PRLR)2 transient complex. 2,276 transient-complex configurations are used for calculating the histograms. Vertical dashed lines indicate γ and ϕ values in the native complex. The two peaks in the γ histogram, to the right and left of the native value, correspond to R2 configurations forming contact mainly with the N-terminal tail and with the rest of the cytokine, respectively. (TIF) [file pcbi.1002427.s003.tif]

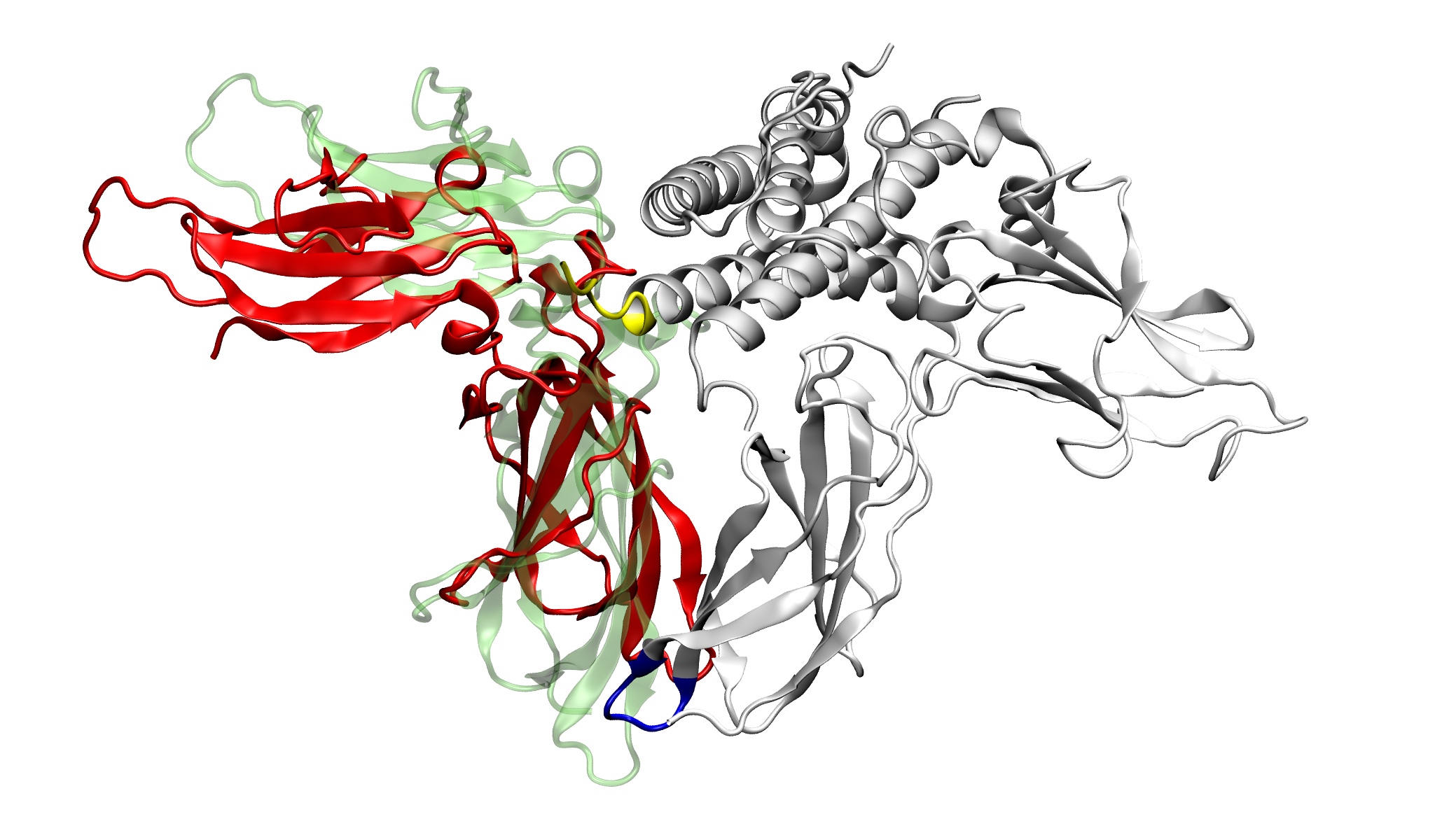

Supplement: Figure S4 — The role of a loop in the C1 subdomain in determining the asymmetric distribution of the transient-complex ensemble. This loop, illustrated here in blue on the GH-GHR system, is between strands A and B. GH and R1 are in gray; native R2 is in lime green; and a R2 configuration in the transient complex is in red. An R2 configuration with a ϕ angle lower than the native value would have its C2 subdomain positioned toward the foreground of the present view and would likely clash with the C1 loop. In contrast, the R2 configuration shown in red has a ϕ angle higher than the native value, and the C2 subdomain, positioned toward the background, is opposite to a flat surface of C1. The extended N-terminal (in yellow) of GH “attracts” R2 configurations (such as the one shown in red) with γ angles higher than the native value. (TIF) [file pcbi.1002427.s004.tif]

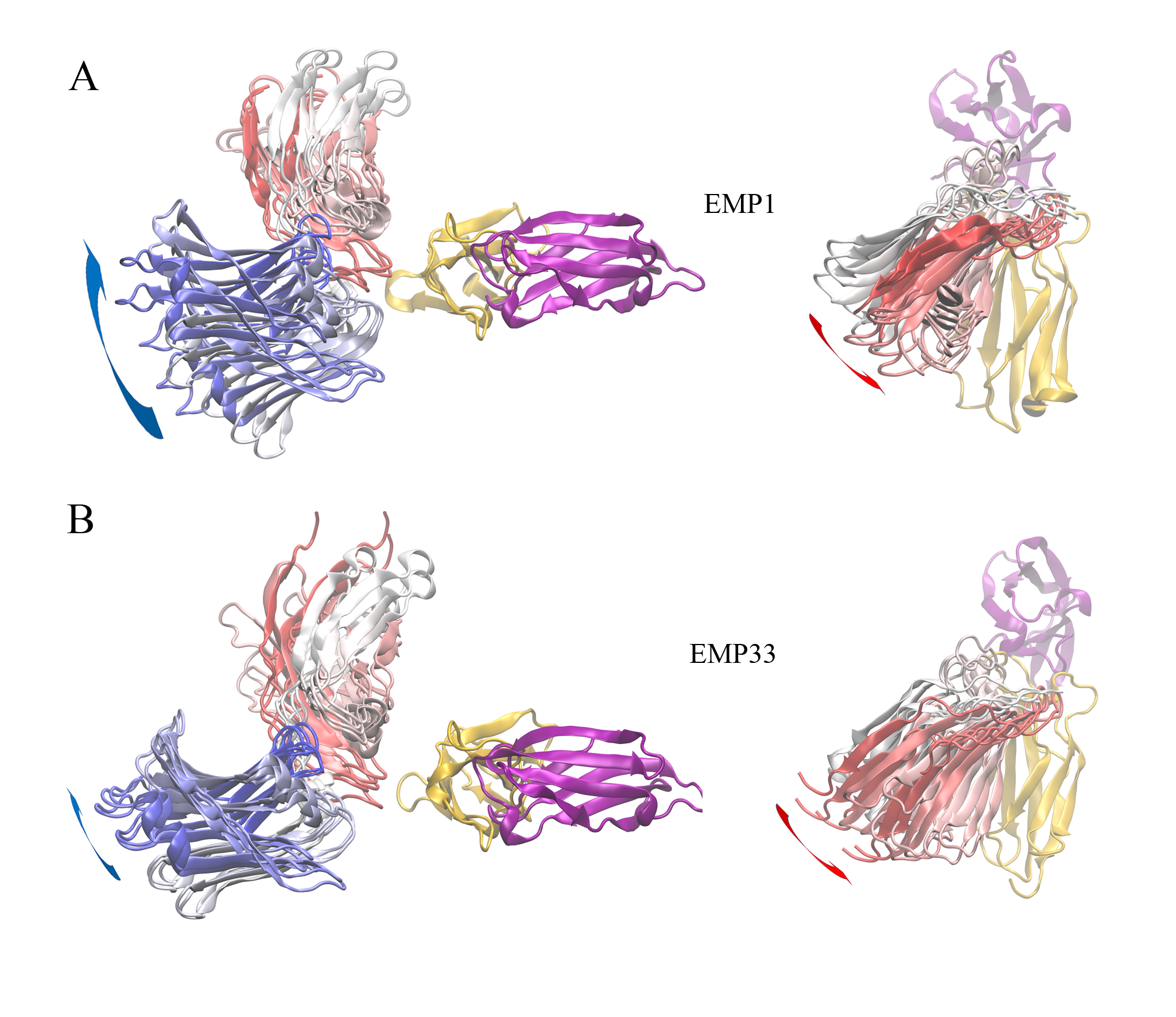

Supplement: Figure S5 — Representative configurations of the transient complexes of two EPO mimetic peptides. (A) EMP1:(EPOR)2. (B) EMP33:(EPOR)2. (TIF) [file pcbi.1002427.s005.tif]

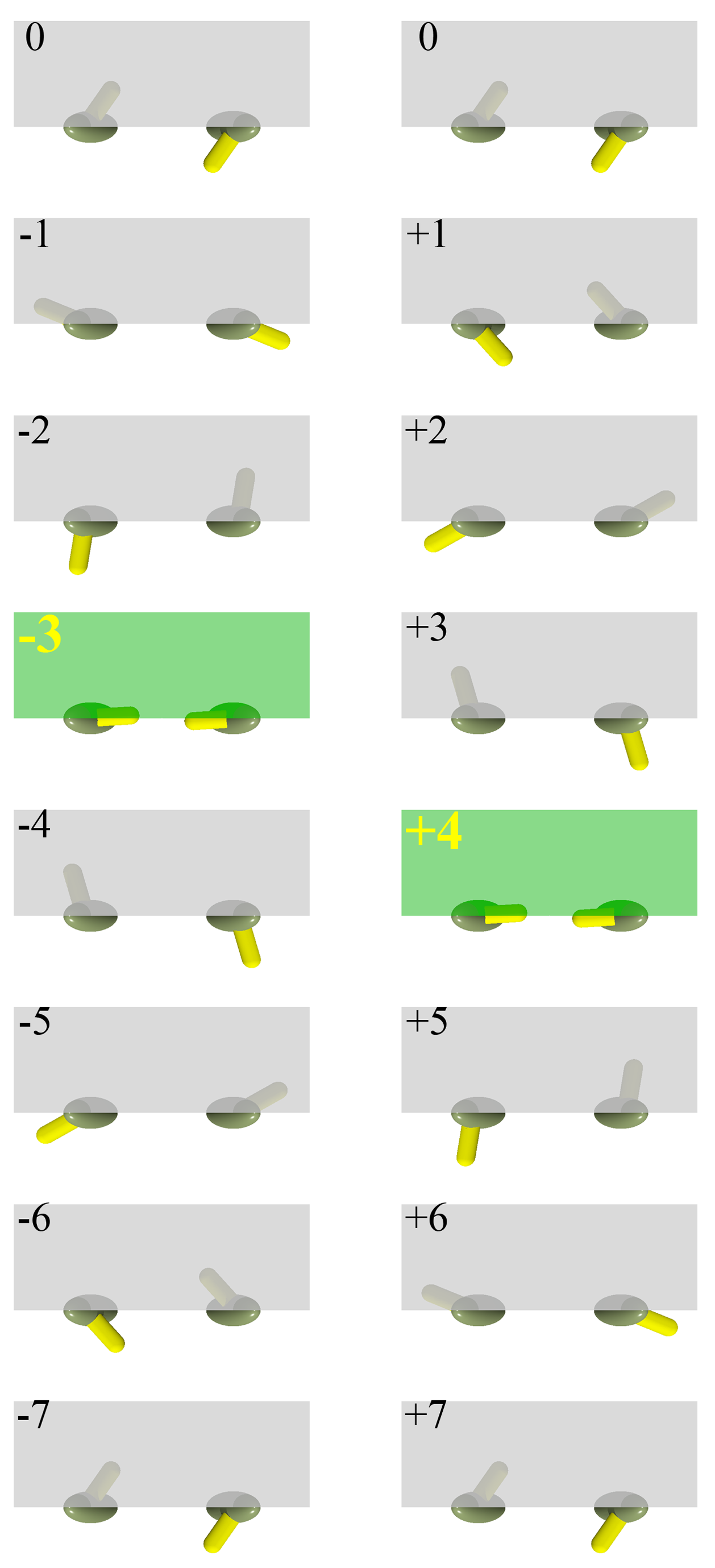

Supplement: Figure S6 — Reorientation of JAK2s resulting from deleting or inserting TMH residues. Top view of the ICDs and the associated JAK2s are shown; a shaded rectangle represents the cell membrane. An α-helix has ∼3.5 residues per turn, so each residue spans ∼360°/3.5 = 103° of the helical wheel. (Left) Deleting each TMH residue would rotate the associated JAK2 counterclockwise for 103°; (Right) Inserting each TMH residue would rotate the associated JAK2 the same amount but in the opposite direction. Negative and positive numbers indicate the total numbers of deleted and inserted TMH residues. Shown in highlight are the three-residue deletion and four-residue insertion, both of which orient the two JAK2 in proximity, ready for transphosphorylation. (TIF) [file pcbi.1002427.s006.tif]

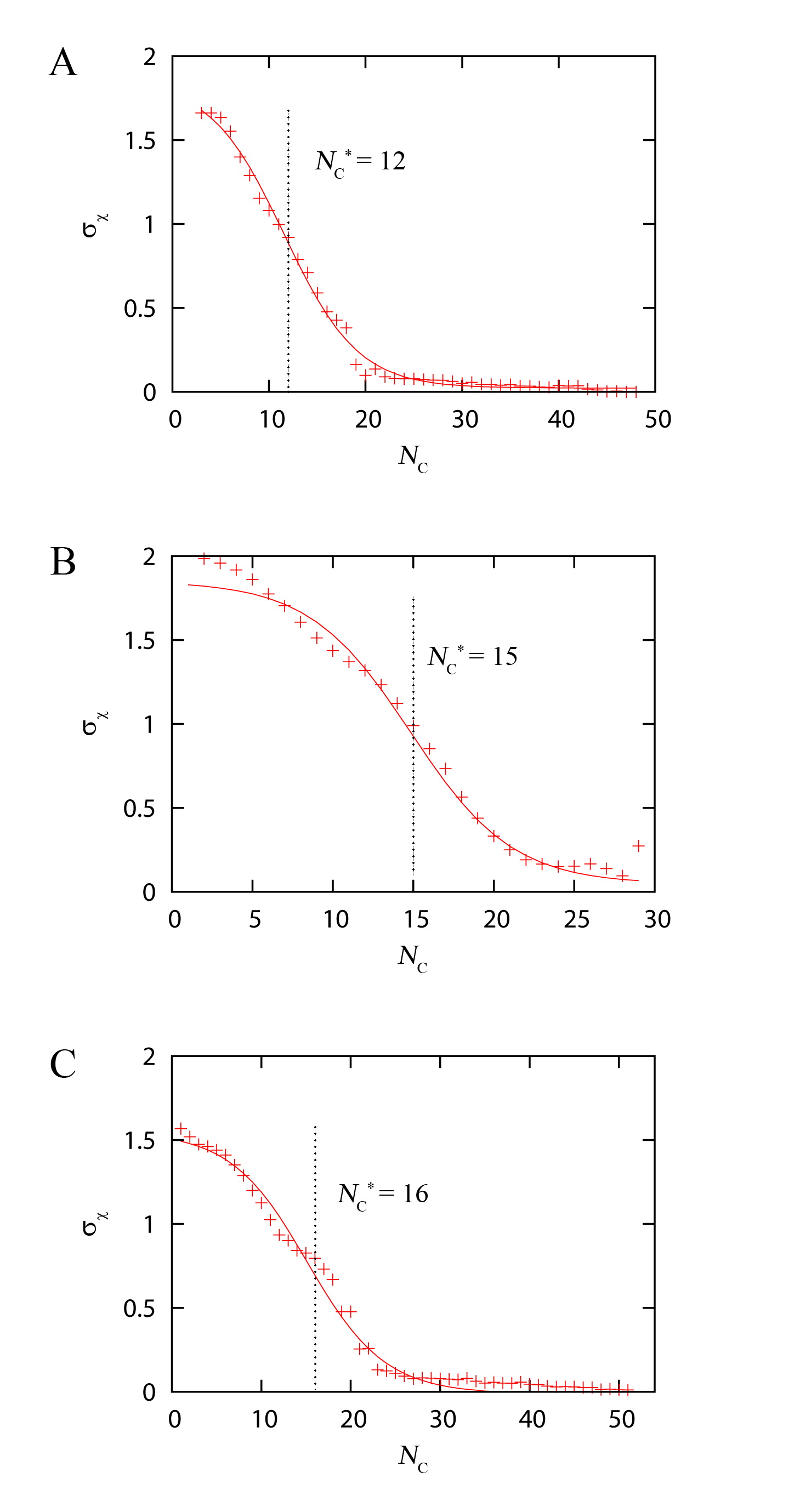

Supplement: Figure S7 — Locating the transient complex. (A) GH:(GHR)2. (B) EPO:(EPOR)2 (C) PRL:(PRKR)2. σχ represents the standard deviation of the χ angles sampled by the clash-free configurations at a given N c. Symbols represent raw data from the randomly generated clash-free configurations; curve represents the fit to a function used for modeling protein denaturation data as two-state transition. The “baseline” with low σχ (and high N c) correspond to configurations in the native-complex well. The “baseline” with high σχ (and low N c) correspond to the start of the unbound state. The midpoint of the transition, where N c is designated N c*, identifies the transient complex. (TIF) [file pcbi.1002427.s007.tif]
