# Supplementary material for: A Common Model for Cytokine Receptor Activation: Combined Scissor-Like Rotation and Self-Rotation of Receptor Dimer Induced by Class I Cytokine
Source: PLoS Comput Biol. 2012 Mar 8;8(3):e1002427. doi: 10.1371/journal.pcbi.1002427 (PMC3297564; doi:10.1371/journal.pcbi.1002427)
Supplement: Text S1 — Methods for calculating association rate constants in solution and results. (DOC) [file pcbi.1002427.s008.doc]

**SUPPORTING TEXT S1**

**Rate constants for forming 1:2 complexes in bulk solution**

Bernat et al. [1] and Broutin et al. [2] have measured the rate constants for binding to the 1:1 complex by R2 ECD coming from the bulk solution for the GH-GHR and PRL-PRLR systems. These measurements motivated us to calculate the rate constants in bulk solution.

According to the transient-complex theory [3], two subunits approach each via translational and rotational diffusion to form the transient complex. Subsequently orientational rearrangement, tightening of the binding interface, and precise fit of the native contacts lead to the native complex. When the second sub-step involves only limited conformational changes of the subunits, then the overall association process is likely to be rate-limited by the first, diffusional sub-step. The association rate constant can then be calculated as

$$k_{\text{a}}=k_{\text{a0}}\exp(-\Delta G_{\text{el}}^{*}/k_{\text{B}}T)$$

where *k*_a0_ is the basal rate constant for reaching the transient complex by unbiased diffusion. The magnitude of *k*_a0_ is determined by the restraints on the relative orientation between the subunits in the transient-complex ensemble. Long-range electrostatic interactions can significantly influence the association rate constant, and is captured by the Boltzmann factor $\exp(-\Delta G_{\text{el}}^{*}/k_{\text{B}}T)$ [4]. In contrast, short-range interactions have little influence on the diffusional approach to form the transient complex (analytical results on model systems show that when the range of the interaction potential is reduced, its influence on the association rate decreases drastically [4]), and are not explicitly accounted for in our theory.

The calculated basal rate constants (*k*_a0_) for the GH-GHR ECD, PRL-PRLR ECD, and EPO-EPOR ECD systems were (1.41 ± 0.04) × 10^5^, (1.55 ± 0.04) × 10^5^, and (3.12 ± 0.06) × 10^5^ M^–1^s^–1^, respectively. The electrostatic interaction energies ($\Delta G_{\text{el}}^{*}$) were 1.0 ± 0.6, –0.5 ± 1.9, and –1.0 ± 1.3 kcal/mol, indicating very modest electrostatic contributions. This is consistent with the mixed charge distributions across the site-2 interfaces of the 1:2 complexes. In comparison, the site-1 interfaces of the 1:1 complexes feature strong charge complementarity for the EPO-EPOR ECD system but mixed charge distributions for the GH-GHR ECD and PRL-PRLR ECD systems, explaining a 5000-fold variation of association rate constants among them [5]. In any event, it appears that all the three systems reach their 1:2 transient complexes largely by unbiased diffusion, and the basal rate constants provide reasonable estimates of the overall association rate constants. These results can be compared with the experimental values, 2.5 × 10^5^ M^–1^s^–1^ for the GH-GHR ECD system [1] and 0.1 × 10^5^ M^–1^s^–1^ for the PRL-PRLR ECD system [2]. No information on experimental errors was given in either case, but the technique used in these studies, surface plasmon resonance, is known to have systematic errors [6,7]. Considering the shortcomings on both the computational side and the experimental side, the agreement in *k*_a_ is reasonable.

In the cellular environments, R2 likely comes from a membrane-bound, preformed receptor dimer. Binding within a preformed dimer largely bypasses the 2-dimensional diffusional step to bring the membrane-bound receptors together, and can be expected to be faster than what is in the situation calculated here (i.e., binding from the bulk solution). The fast binding of R2 ensures that each bound cytokine molecule leads to the 1:2 activation complex before it has a chance to unbind

**Implementation of the transient-complex theory for *k*_a_ calculation**

The association rate constant *k*_a_ involves two components, *k*_a0_ and $\Delta G_{\text{el}}^{*}$. The basal rate constant *k*_a0_ concerns reaching the transient complex by unbiased diffusion, and was obtained by Brownian dynamics simulations in which no force or torque was present to bias the translational and rotational diffusion.

The electrostatic interaction energy $\Delta G_{\text{el}}^{*}$ was obtained from solving the full Poisson-Boltzmann equation, by the Adoptive Poisson-Boltzmann Solver (APBS version 1.2) [8], on 100 transient-complex configurations randomly selected from the transient-complex ensemble. The protein atoms were assigned AMBER charges [9] and Bondi radii [10]. The protonation states of titratable groups were assigned for a nominal pH of 7, with the N-terminal, Lys and Arg residues protonated, and the C-terminal, Asp and Glu residues unprotonated (pH values in the experimental studies of Bernat et al. [1] and Broutin et al. [2] were not reported). The dielectric constant of the protein solute was set to 4, and the dielectric constant of the solvent was set to 78.5, corresponding to a temperature of 300 K. The dielectric boundary was specified as the van der Waals surface [3] by setting the *srfm* flag to *mol* and *srad* to 0. The ionic strength was 160 mM, typically used in surface plasmon resonance experiments (buffer conditions were not reported by Bernat et al. [1] and Broutin et al. [2]). For each transient-complex configuration, the electrostatic interaction energy Δ*G*_el_ between R2 and the 1:1 complex was calculated as

$$\Delta G_{\text{el}}=G_{\text{el}}(\text{TC})-G_{\text{el}}(\text{R}2)-G_{\text{el}}(1:1)$$

where the three terms on the right-hand side denotes the electrostatic free energies of the transient-complex configuration, R2, and 1:1 complex, respectively. The average of Δ*G*_el_ over the 100 transient-complex configurations produced $\Delta G_{\text{el}}^{*}$.

**REFERENCES**

1. Bernat B, Pal G, Sun M, Kossiakoff AA (2003) Determination of the energetics governing the regulatory step in growth hormone-induced receptor homodimerization. Proc Natl Acad Sci USA 100: 952-957.

2. Broutin I, Jomain JB, Tallet E, van Agthoven J, Raynal B, et al. (2010) Crystal structure of an affinity-matured prolactin complexed to its dimerized receptor reveals the topology of hormone binding site 2. J Biol Chem 285: 8422-8433.

3. Alsallaq R, Zhou HX (2008) Electrostatic rate enhancement and transient complex of protein-protein association. Proteins 71: 320-335.

4. Zhou HX (1997) Enhancement of protein-protein association rate by interaction potential: accuracy of prediction based on local Boltzmann factor. Biophys J 73: 2441-2445.

5. Pang X, Qin S, Zhou HX (2011) Rationalizing 5,000-fold differences in receptor-binding rate constants of four cytokines. Biophys J 101: 1175-1183.

6. Schreiber G, Haran G, Zhou H-X (2009) Fundamental aspects of protein-protein association kinetics. Chem Rev 109: 839-860.

7. Qin S, Pang X, Zhou HX (2011) Automated prediction of protein association rate constants. Structure 19: 1744-1751.

8. Baker NA, Sept D, Joseph S, Holst MJ, McCammon JA (2001) Electrostatics of nanosystems: Application to microtubules and the ribosome. Proc Natl Acad Sci USA 98: 10037-10041.

9. Cornell WD, Cieplak P, Bayly CI, Gould IR, Merz KM, et al. (1995) A second generation force field for the simulation of proteins, nucleic acids, and organic molecules. J Am Chem Soc 117: 5179-5197.

10. Bondi A (1964) van der waals volumes and radii. J Phys Chem 68: 441-451.
